# A Health Officer for Atlanta

**Published:** 1901-04

**Authors:** 


### EDITORIAL NOTES AND COMMENTS.

The Business office of THE JOURNAL-RECORD is No. 64 Marietta Street.

The Editorial office is 318-319-320 The Prudential.

Address all Business communications to Dr. M. B. Hutchins, Mgr.

Make remittances payable to THE ATLANTA JOURNAL-RECORD OF MEDICINE.

On matters pertaining to the Editorial and Original Communications address Dr. Bernard Wolff, Atlanta.

Reprints of original articles will be furnished at cost price. Requests for the same should always be made *on the manuscript*.

We will present, post-paid, on request, to each contributor of an original article, twenty (20) marked copies of THE JOURNAL-RECORD containing such article.

### A HEALTH OFFICER FOR ATLANTA.

AT the time that the question was agitated of appointing a health officer for Atlanta a somewhat acrimonious discussion arose between some of the local physicians over the feasibility and desirability of creating such an office. It was contended on the one side that such an official had been rendered necessary by the growth of the city and the consequent increase in the amount of communicable disease, while the other side maintained that the duties required of a health officer did not agree with their conception of professional privilege, and that they would occasion an intolerable intrusion upon the physician's rights in relation to his patient. The discussion never drew to a conclusion. Lately a medical member of the city council has introduced a resolution before that body to establish the office and appoint an official whose duty shall cover the general supervision of contagious and infectious disease so far as concerns its spread. The measure seems as yet to be on the knees of the gods.

As for the main points at issue, there can be no doubt but that

Atlanta has grown to be too tight a fit for village methods, and that there is definite need for a recasting of the system for protection against communicable disease which obtained in the earlier stages of the city's development. The safety of its citizens and the peace of mind of those who have children demand and require as thorough and efficient protection against contagious and infectious disease as can be gotten out of our present knowledge and facilities. This much is self-evident and does not admit of discussion. The best and most expedient means of carrying out this object might allow of difference of opinion.

The method generally pursued in large cities is for this work to be under the control of a bureau of contagious disease connected with the board of health, and which consists of a chief inspector and as many assistants as are needed. When a case of contagious disease is reported to the bureau, an inspector is sent, who examines the patient with the attending physician, agrees or not with his diagnosis and gives such recommendations as his duty requires. The inspector does not interfere with the plan of treatment adopted by the attending physician, nor does he do anything prejudicial to the interest of the latter in relation to the patient or family. He is merely a paid official, impersonal in attitude. Far from interfering with the physician's prerogative or compromising his position as the family practitioner, the health officer is of great aid to him and often relieves him from responsibilities that are as irksome as difficult. If the health officer is a man of approved competence and experience, free from venality and politics, there is no one in a community more useful or more needed by the public and the profession. It is necessary that he possess these qualities; otherwise it may readily be seen how he could become an intolerable nuisance.

ONE G. K. Woodward was tried before Judge Reid of the city court on the charge of illegal practice of medicine. His defense was that he was in the employment of a medical concern of which a regularly licensed physician and druggist was the head. He claimed that he did not believe in medicine, and that he used massage, electricity and "ointment" for the cure of disease. On these grounds he secured a verdict of acquittal. This trial was investigated by the Atlanta Society of Medicine, through its attorney, Mr. W. W. Visanska, who, in spite of this temporary backset, intends to wage a determined fight against illegal practitioners in this city. It is a difficult task but a by no means impossible one. The chief difficulty is, not in collecting sufficient incriminating evidence, but in finding a jury who will convict on it. The average jury seems controlled by the sentiment, "give the poor devil a chance" rather than by the plain fact that the "poor devil" is an offender against a written law. Another obstacle in the way of justice is the character and position of the quack's patients or dupes. They are frequently people of prominence and influence, whose testimony in favor of the quack carries great weight before the jury and often secures acquittal in the face of evident guilt. The mass of laymen are prone to think that the members of the regular medical profession endeavor to suppress quackery from motives of self-interest—to be rid of formidable rivals who threaten their source of livelihood—and it assumes that in upholding charlatanry it is taking sides with the "under dog" against its stronger adversary. It is well-nigh idle to attempt to convince the unthinking of the groundlessness of this belief, or to explain to them that opposition to quackery is aroused from no selfish reason on the part of the medical profession, but for the protection of the public against an evil that assails them, and which they do not or will not understand and appreciate. Whether or not the public is open to conviction on this point, or will lend more than a jeering attention to it, it is the classic duty of physicians to protect the unwary and the ignorant against fraud and deception appearing under the guise of friendliness and philanthropy, and to search out and expose quackery and pretense in all their various forms. Fortified by these high motives, the Atlanta Society of Medicine has no thought of relinquishing the fight, and will continue to push it by every means in its power until the city is freed from this "botch of Egypt."

---

FOUR physicians of this city were paid $190 each for inspecting smallpox cases. The Council reconsidered their action and requested the money to be refunded. This decision was finally reversed and the money was repaid the doctors. It would be prudent for physicians who are called upon to do municipal work to be sure of their pay beforehand, as the City Council displays an habitual reluctance in paying doctor's bills.

---

THE King's Daughters have opened a small hospital for incurables on Church street. The resident physician is Dr. S. A. Visanska. The hospital can accommodate ten patients. The Presbyterians of Atlanta contemplate the erection in the near future of a hospital. They are actively at work securing funds to carry out the purpose. The hospital will be non-sectarian.

---

REMEMBER that the State Association meets at Augusta, April 17, 18, 19. Send the title of your paper to Dr. J. B. Graham, Savannah, Ga., and get a good position on the program.

---

THE selection in the March number, "Sequelæ of Scarlet Fever," should have been credited to the *Medical Council.* Our apologies are tendered that valued exchange for the oversight.

